# Relationships Between Food Groups and Eating Time Slots According to Diabetes Status in Adults From the UK National Diet and Nutrition Survey (2008–2017)

**DOI:** 10.3389/fnut.2021.692450

**Published:** 2021-09-30

**Authors:** Chaochen Wang, Suzana Almoosawi, Luigi Palla

**Affiliations:** ^1^Department of Public Health, Aichi Medical University, Nagakute, Japan; ^2^Faculty of Medicine, School of Public Health, Imperial College London, London, United Kingdom; ^3^Department of Public Health and Infectious Diseases, University of Rome La Sapienza, Rome, Italy; ^4^Department of Medical Statistics, London School of Hygiene & Tropical Medicine, London, United Kingdom; ^5^Department of Global Health, School of Tropical Medicine and Global Health, University of Nagasaki, Nagasaki, Japan

**Keywords:** chrononutrition, time of eating, the UK national diet and nutrition survey, nutrition epidemiology, correspondence analysis, diabetes

## Abstract

Time of eating is associated with diabetes and obesity but little is known about less healthy foods and specific time of their intake over the 24 h of the day. In this study, we aimed to identify potential relationships between foods and their eating time and to see whether these associations may vary by diabetes status. The National Diet and Nutrition Survey (NDNS) including 6,802 adults (age ≥ 19 years old) collected 749,026 food recordings by a 4-day-diary. The contingency table cross-classifying 60 food groups with 7 pre-defined eating time slots (6–9 a.m., 9 a.m.–12 p.m., 12–2 p.m., 2–5 p.m., 8–10 p.m., 10 p.m.–6 a.m.) was analyzed by Correspondence Analysis (CA). CA biplots were generated for all adults and separately by diabetes status (self-reported, pre-diabetes, undiagnosed-diabetes, and non-diabetics) to visually explore the associations between food groups and time of eating across diabetes strata. For selected food groups, odds ratios (OR, 99% CI) were derived of consuming unhealthy foods at evening/night (8 p.m.–6 a.m.) vs. earlier time in the day, by logistic regression models with generalized estimating equations. The biplots suggested positive associations between evening/night and consumption of puddings, regular soft drinks, sugar confectioneries, chocolates, beers, ice cream, biscuits, and crisps for all adults in the UK. The OR (99% CIs) of consuming these foods at evening/night were, respectively, 1.43 (1.06, 1.94), 1.72 (1.44, 2.05), 1.84 (1.31, 2.59), 3.08 (2.62, 3.62), 7.26 (5.91, 8.92), 2.45 (1.84, 3.25), 1.90 (1.68, 2.16), and 1.49 (1.22, 1.82) vs. earlier time in the day adjusted for age, sex, body mass index (BMI), and social-economic levels. Stratified biplots found that sweetened beverages, sugar-confectioneries appeared more strongly associated with evening/night among undiagnosed diabetics. Foods consumed in the evening/night time tend to be highly processed, easily accessible, and rich in added sugar or saturated fat. Individuals with undiagnosed diabetes are more likely to consume unhealthy foods at night. Further longitudinal studies are required to ascertain the causal direction of the association between late-eating and diabetes status.

## Introduction

The timing of energy intake is associated with obesity and diabetes (1). Specifically, eating late at night or having a late dinner was found to be related to a higher risk of obesity (2, 3), hyperglycemia (4), metabolic syndrome (5), diabetes (6), and poorer glycemic control among diabetics (7). However, the relationship between food choice and the time of food consumption during the day is left largely unknown. Shift workers have an increased risk of obesity (8, 9) and diabetes (10), possibly due to the limited availability of healthy food choices during their night shifts (8, 11). Previous survey data from the UK National Diet and Nutrition Survey Rolling Programme (NDNS RP) found that overall, 3.4% of men and 2.3% of women aged 19–64 had fasting glucose concentrations above the clinical cut-off for diabetes (⩾ 7 mmol/L). In addition, the proportion of men with undiagnosed diabetes increased with age to over 20% in the UK population (12). Identifying those unhealthy foods that might be chosen during late-night time would be helpful when guiding people to change their eating habits for either weight loss or glycemic control. Dietary diary recordings from NDNS RP surveys can provide detailed food choice data for exploration of the relationships between food groups and their time of consumption in the general population.

In this study, we aimed to describe the relationship between food groups and the time of day when they were consumed, and how such relationships may vary by the status of type 2 diabetes using the data published by the NDNS RP from 2008 to 2017 as this survey includes diet diaries providing detailed information on the time of day of food intake.

## Methods

6,802 adults (2,810 men and 3,992 women) and 749,026 food recordings collected by the NDNS RP 2008-17 were analyzed in the current study (13). The survey comprised a cross-section representative sample of the UK adult population taken over the period 2008-2017. The sample was randomly drawn from a list of all addresses in the UK, clustered into postcode sectors. Details of the rationale, designs, and methods of the survey can be found in the previously published official study reports (14, 15). The NDNS-RP, funded by Public Health England and the UK Food Standards Agency, is registered with the ISRTCN registry in study ID ISRCTN17261407 and received ethical approval from the Oxfordshire Research Ethics Committee.

A 4-day food diary method was used in the NDNS RP to collect the detailed food items and their time of consumption from participants. Comparison between the food diary method and a repeated 24-h recall questionnaire was performed in a subset of the study sample prior to the launch of the NDNS RP in 2008 and found that they were similar in terms of response rate as well as the ability to collect correct nutrition intake data. The 4-day food diary method was adopted because it is considered to be more flexible and adaptable to cover a wide population age range in the survey. More details can be found in the Appendix A of the official NDNS RP study report (14, 15). Furthermore, the same food diary methods are actually used in large studies conducted in the UK, such as the Medical Research Council (MRC) National Survey of Health and Development (NSHD) (1946 British Birth Cohort) (16), the EPIC Norfolk Study (17), the UK Women's Cohort Study in Leeds (18), and the Avon Longitudinal Study of Parents and Children (ALSPAC) cohort (19). Another validation study of the food records against double-labeled water had also been undertaken among a subset of NDNS participants. Full results of the analysis have been reported in Appendix X of the official survey report (20).

In the food diary recordings, time of the day was categorized into 7 slots: 6–9 a.m., 9–12 noon, 12–2 p.m., 2–5 p.m., 5–8 p.m., 8–10 p.m., and 10 p.m.–6 a.m. Foods recorded were classified into 60 standard food groups with 1 to 10 subgroups each: the details are given in Appendix R of the NDNS official report (21). We focused on the 60 standard food groups in the current analysis. Diabetes status was defined as: (1) healthy if fasting glucose was lower than 6.10 (mmol/L), hemoglobin A1c (HbA1c) was less than 6.5 (%), and without self-reported diabetes or treatment for diabetes (*n* = 2,626); (2) pre-diabetic if fasting glucose was between 6.10 and 6.99 (mmol/L, inclusive) but without self-reported diabetes and without treatment for diabetes (*n* = 133); (3) undiagnosed diabetic if either fasting glucose was higher or equal to 7.00 (mmol/L) or HbA1c higher or equal to 6.5 (%) but without self-reported diabetes and treatment for diabetes (*n* = 99); (4) diabetic if the participant had self-reported diabetes or was under treatment for diabetes (*n* = 227). There was also a large number of adults (3,717 adults of whom 1,519 men and 2,198 women) whose diabetes status could not be confirmed due incomplete variable information; these were retained in the whole sample (unstratified) analyses. In addition, the National Statistics Socio-economic Classification (22) was applied in the survey and, accordingly, the socio-economic status of participants was classified in one of 8 categories.

Correspondence analysis (CA) (23–25) was used as a tool for data mining, visualization, and hypotheses generation using half of the randomly selected NDNS diary entries data. Specifically, the contingency table was generated by cross-tabulating 60 food groups and 7 time slots. Through CA, the 60 categories of standard foods and the 7-time slots were projected on biplots, i.e., onto two-dimensional plots that could jointly display large percentage of the Chi-Square deviation (or inertia) of the contingency table. Biplots that graphically show the association between time of day and food groups were derived for all adults and separately according to their diabetes status.

CA is a statistical technique to explore relationships between categorical variables in a two-dimensional contingency table. In the current analysis context, CA was used as a tool to visually depict the relationship between food groups and time of consumption. CA allowed us to identify whether food groups have a similar or different “profile” across time categories or, symmetrically, whether times of day have a similar or different “profile” across food groups. In particular, “profile” indicates the relative frequency of the consumption of one food across different times in the day (or, symmetrically, the relative frequency of consumption of different foods at one specific time slot) and what CA measures is its departure from the average food (or time of day) profile. One simple example is that if about 77.8% of all foods were consumed during the daytime (earlier than 8 pm), but only 23.5% of beer consumption were recorded during the daytime, then we say beer has a “profile” different from the average food profile with respect to time of day of consumption and, in particular, beer is associated to evening/night consumption. CA can produce biplots to visually show the χ^2^ deviation of food (and time) profiles from the average profile which is called “inertia.” These biplots use the first two most informative dimensions to display the inertia of the contingency table. The horizontal axis of the biplot represents the direction along which the contingency table rows and columns show their greatest deviations from the average row or column profile. The vertical axis represents the direction, perpendicular to the first, having the second-largest deviations. There are two percentage labels for each axis, indicating how much of the total inertia were explained along that axis. The sum of the two percentages is lower than 100%, the remaining inertia cannot be shown when reducing to two-dimensions if there are more than three foods or time-slots. The origin in each biplot is the average profile of all points in the plot, while the length of the vector from the origin to each profile point represents its deviation from the average profile. The distance between row (food) and column (time slots) profile points and the direction in which they lie away from the origin is indicating how they are associated with each other. The potential association is greater if (1) points are located in similar directions away from the origin and (2) the farther they are from the origin.

To account for the hierarchical structure of the data (food recorded by the same individuals who lived within the same area/sampling units) and to calculate population average odds ratios (OR), logistic regression models with generalized estimating equations (GEE) were subsequently used to test the associations that were first suggested by visual inspection of biplots generated by CA, using the remaining half of the diary entries data. The marginal ORs and their 99% CI were derived of consuming unhealthy food groups (selected by CA) later in the day (8 p.m.–6 a.m., i.e., in the evening and night) compared with earlier in the day (in the morning or afternoon). In the fixed effect of the logistic regression models, 2-time slots and 4 diabetes statuses were entered with interaction terms. This was done to assist performing post fitting estimations of OR for each diabetes status using the same model and avoid running more models on smaller datasets with less statistical power as well as the risk of multiple testing. CA and biplots were conducted and generated by the following packages under R environment (26): FactoMineR, factoextra, ggplot2, and ggrepel (27–30). Logistic regression models with GEE were performed with SAS procedure GENMOD (31) adjusted for age, sex, body mass index (BMI) and socio-economic levels, which were deemed the main potential confounders of the associations.

## Results

The dataset consisted of 2,810 (41.3%) men and 3,992 (58.7%) women aged older than or equal to 19-year-old with a mean age of 49.9 years (*SD* = 17.6). Of these individuals, 22.6% were current smokers, and 24.3% were past smokers. The average BMI was 27.7 kg/m^2^ (*SD* = 5.41). Among the food recordings collected (*n* = 749,026), 56.9% were recorded during traditional breakfast (6 a.m.–9 a.m.: 14.3%), lunch (12 noon–2 p.m.: 18.5%), or dinner (5 p.m.–8 p.m.: 24.1%) time slots, more details can be found in Supplementary Table 1. Table 1 shows the top 37 food groups that contributed to 90% of the total calories consumed by adults in NDNS RP. These food groups accounted for 478,028 of the total diary entries (63.8 %). The random process split the whole set of food recordings into a hypotheses generating dataset of 374,682 and a testing dataset of 374,344 entries.

**Table 1:** The top 37 food groups which contributed up to 90% of the total calories by UK adults (NDNS RP 2008–2017) were sorted by decreasing cumulative percentage of calories.

**Food group names**	**n**	**Calories**	**Relative prop (%)**	**Cal prop (%)**	**Cal cum prop (%)**
Pasta & rice and other cereals	18,353	3512069.99	2.45	7.36	7.36
White bread	18,434	3245641.19	2.46	6.80	14.17
Chips, fried and roast potatoes and potato products	6,749	1884058.68	0.90	3.95	18.12
Cakes, buns, sweet pastries, fruit pies	7,806	1710594.27	1.04	3.59	21.70
Vegetable (not raw)	51,317	1665474.02	6.85	3.49	25.19
Biscuits	13,200	1662598.06	1.76	3.49	28.68
Fruit	33,903	1641675.02	4.53	3.44	32.12
Miscellaneous unclassified foods	48,597	1639024.81	6.49	3.44	35.56
Chicken/turkey	8,863	1617820.30	1.18	3.39	38.95
Cheese	10,983	1492015.32	1.47	3.13	42.07
Beer lager	8,199	1484001.20	1.09	3.11	45.19
Semi-skimmed milk	57,611	1302649.72	7.69	2.73	47.92
Potatos other (in salads and dishes)	10,113	1291447.61	1.35	2.71	50.62
Fat spreads	37,960	1215278.60	5.07	2.55	53.17
Beef	4,987	1124560.42	0.67	2.36	55.53
High fiber breakfast cereals	8,215	1072813.73	1.10	2.25	57.78
Whole meal bread	7,193	1070695.89	0.96	2.24	60.02
Chocolate	6,495	1046112.65	0.87	2.19	62.22
Wine	6,967	1027792.96	0.93	2.15	64.37
Brown, granary and wheatgerm bread	6,183	1009074.95	0.83	2.12	66.48
Butter	10,203	965901.11	1.36	2.02	68.51
Eggs	7,554	964769.19	1.01	2.02	70.53
Soft drinks not diet	11,387	940516.516	1.52	1.97	72.50
Reduced fat spreads	12,620	848834.89	1.68	1.78	74.28
Crisps and savory snacks	5,664	835671.58	0.76	1.75	76.04
Sausages	3,025	775004.13	0.40	1.62	77.66
Meat pastries	1,979	744639.89	0.26	1.56	79.22
Bacon and ham	8,467	738727.49	1.13	1.55	80.77
Yogurt	6,776	665484.55	0.90	1.40	82.16
Low-fiber breakfast cereals	4,303	560296.32	0.57	1.17	83.34
Nuts and seeds	6,259	559873.88	0.84	1.17	84.51
Oily fish	2,610	550425.36	0.35	1.15	85.67
Whole milk	13,628	530449.07	1.82	1.11	86.78
White fish, shellfish	1,597	498928.82	0.21	1.05	87.82
Puddings	2,291	459784.62	0.31	0.96	88.79
Other milk cream	6,605	434239.37	0.88	0.91	89.70
Pork	1,832	420503.76	0.24	0.88	90.58

*NDNS RP, National Diet and Nutrition Survey Rolling Programme*.

*Prop: proportion; Cal Prop: calorie proportion; Cal Cum Prop: Calorie cumulative proportion*.

**Figure 1:**
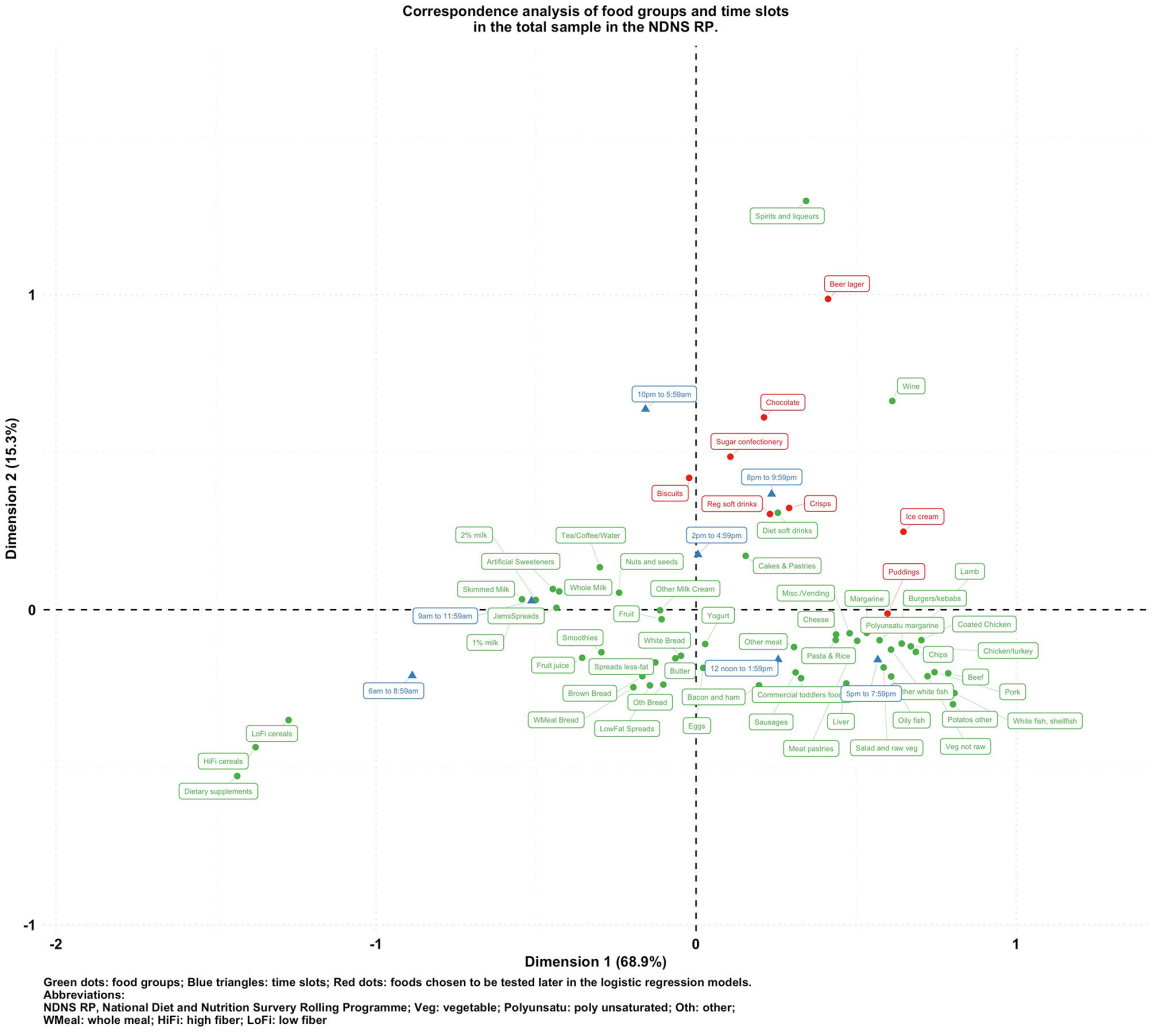
Biplot of food groups and eating time slots in the total sample in the NDNS RP.

**Figure 2:**
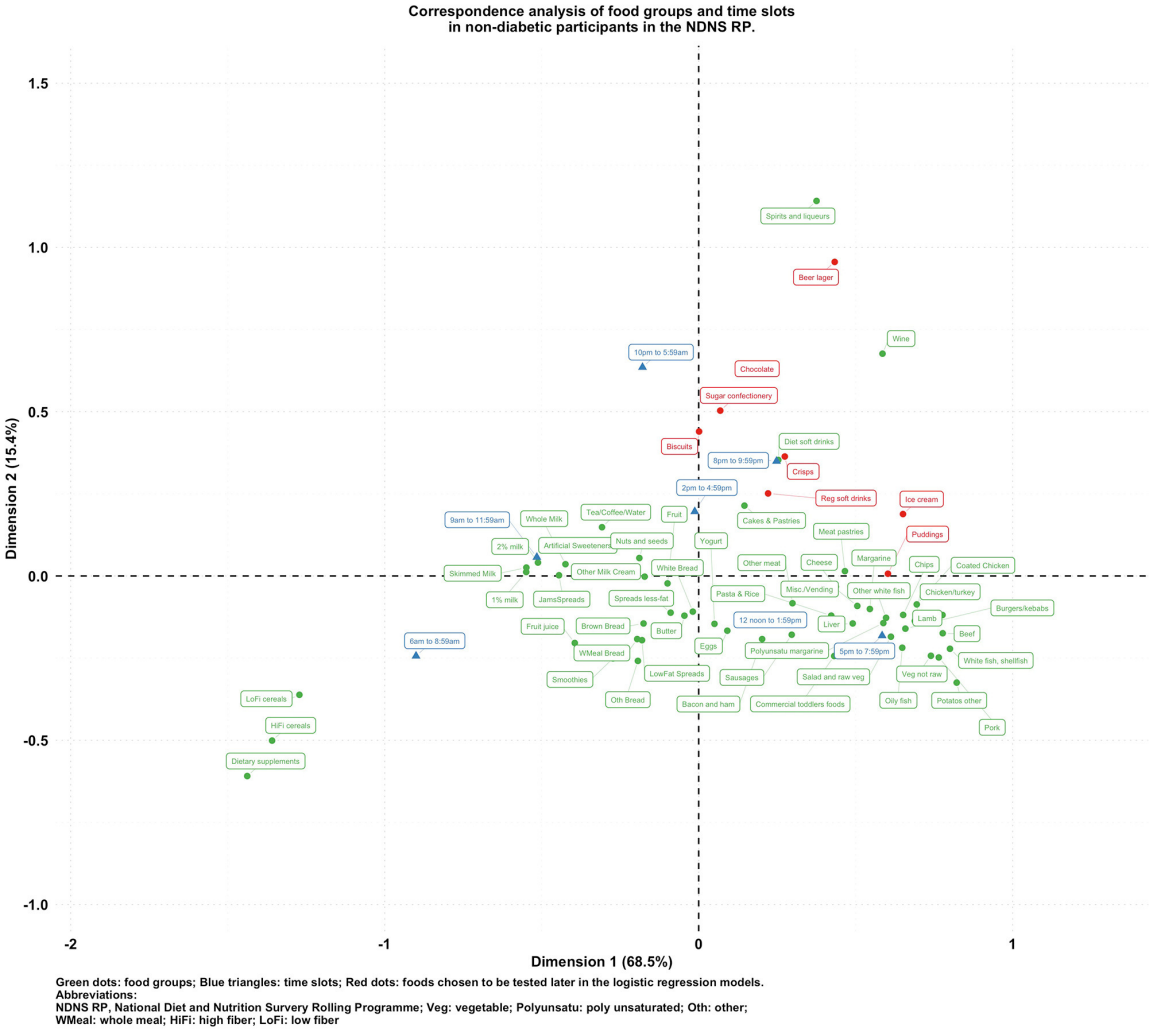
Biplot of food groups and eating time slots in non-diabetic participants in the NDNS RP.

**Figure 3:**
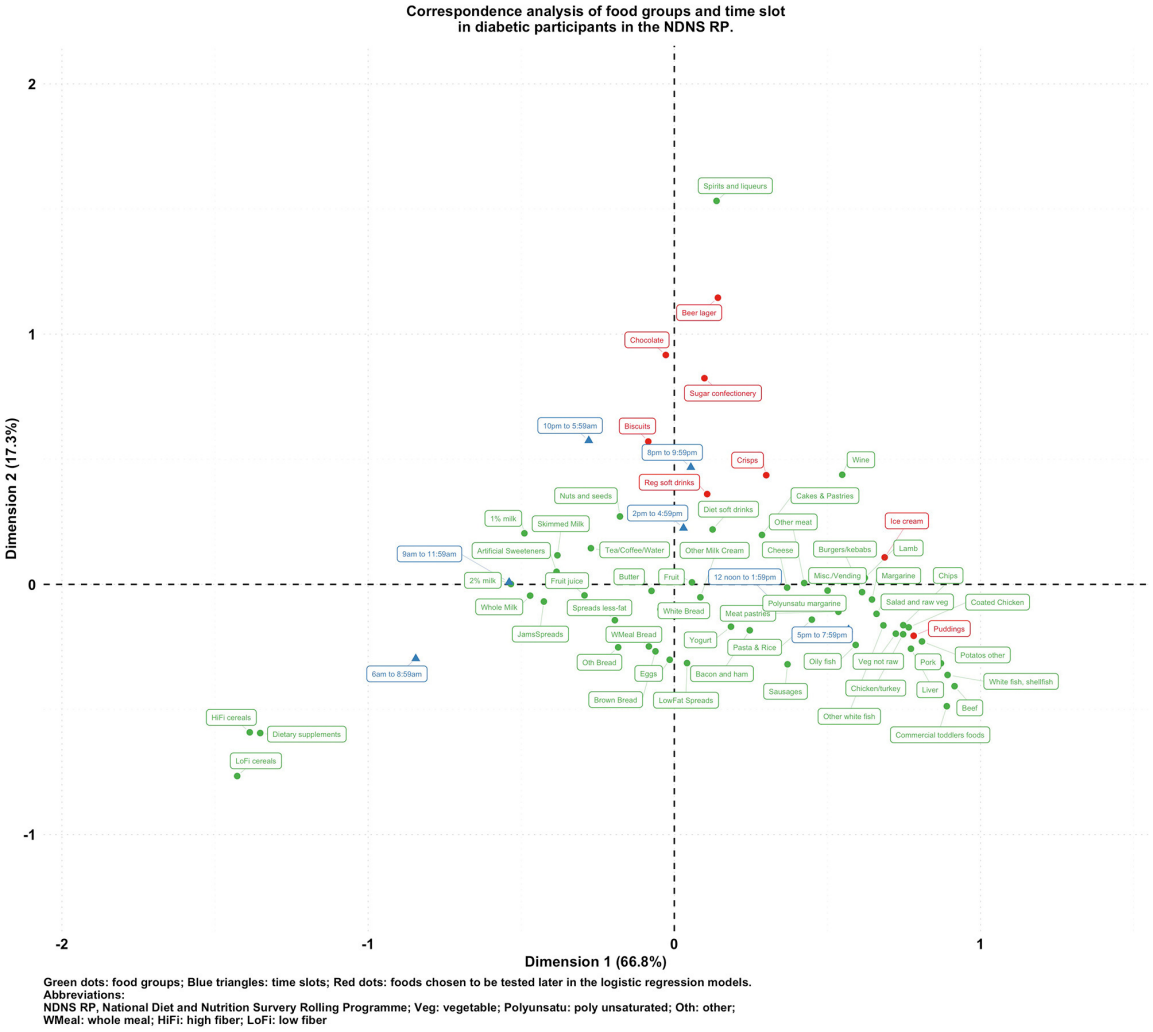
Biplot of food groups and eating time slots in diabetic participants in the NDNS RP.

**Figure 4:**
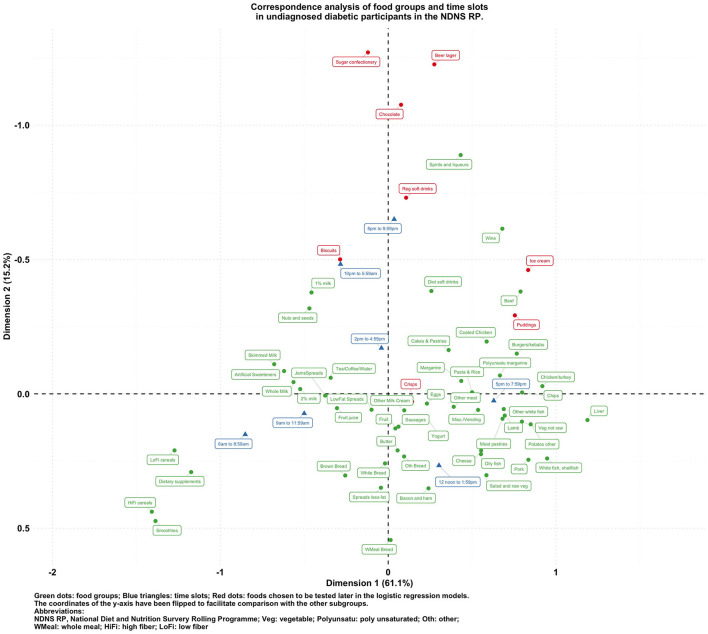
Biplot of food groups and eating time slots in undiagnosed diabetic participants in the NDNS RP.

**Figure 5:**
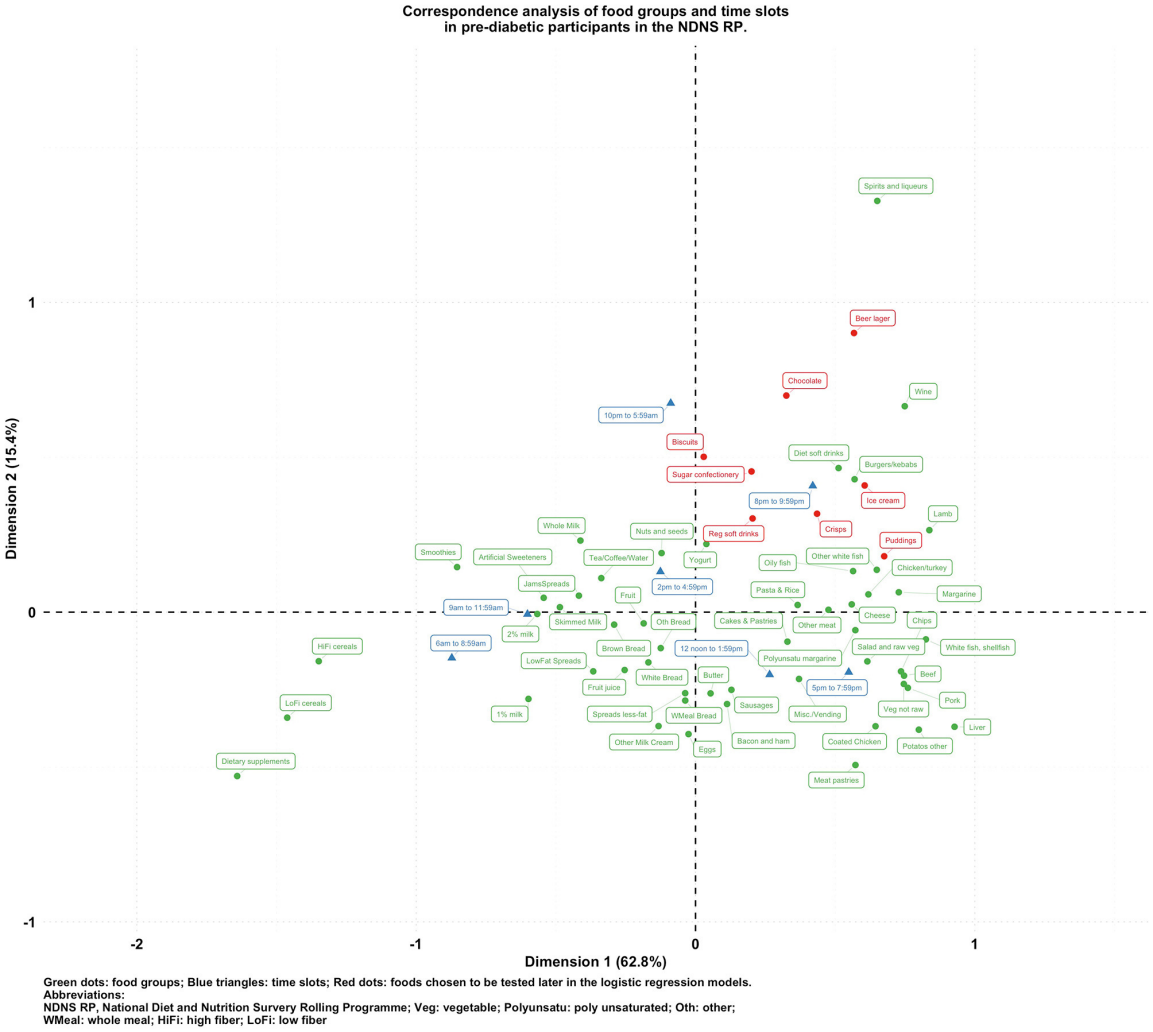
Biplot of food groups and eating time slots in pre-diabetic participants in the NDNS RP.

Figures 1–5 present the CA biplots that visually summarize the associations between 60 food groups and the time of their consumption in the entire sample and then stratifying by their diabetes status. In Figure 1, the horizontal axis explains 68.9% of the association structure (inertia) between foods and time slots while the vertical axis reflects 15.3% of the same relationship. Therefore, a total of 84.2% of the inertia between foods and time slots was captured in this figure which shows a visual summary of how those two categorical variables are related. Specifically, time slots later than 8 p.m. are shown in the upper side of the plot closer to alcoholic products or highly processed/energy-dense foods (sugar confectioneries, chocolates, biscuits, regular soft drinks, ice cream, crisps); times earlier than noon appear in the left-hand side together with typical breakfast foods (cereals, milk, bread, etc.).

To visualize the potentially different associational patterns between food groups and time slots according to diabetes status, Figures 2–5 display the CA biplots in subsets of the data. Depending on diabetes status, these biplots explained between 76.3 and 84.1% of the inertia in the data. Similar to the biplot created from the total sample (Figure 1), later times in the day (8 p.m. and later) are shown in the upper side of each figure and suggested an association with alcoholic beverages and highly processed or energy-dense food groups. Additionally, some food group/time slot associations appeared to vary according to diabetes status. For example, puddings seemed to be closer to a later time in the day among undiagnosed diabetics (Figure 4), while for diagnosed diabetic patients (Figure 3), they were closer to traditional dinner time (5 p.m.–8 p.m.) or earlier in the day. Furthermore, sugar confectioneries/chocolates/biscuits/regular soft drinks appeared to be associated with later times in the day (8 p.m. or later) more strongly among undiagnosed diabetics (Figure 4) than in other subgroups.

Based on the findings suggested from Figures 1–5, we decided to focus on puddings, regular soft drinks, confectioneries, chocolates, beers, ice cream, biscuits, and crisps as these foods either showed a particularly strong association with time of the day or a different pattern of association across different strata of the survey sample; hence, we tested the following null hypotheses using logistic regression models (adjusted for age, sex, BMI, and socio-economic levels) with GEE: that the odds of consuming each selected food at a later time of the day (8 p.m.–6 a.m.) is the same compared to an earlier time in the day; and the associations of the above-mentioned food groups and time slots are the same among participants with different diabetes status (i.e., no interaction between the time of food intake and diabetes status). The results are summarized in Table 2.

**Table 2:** Odds ratio (99% CI) for food groups eaten at night (8 p.m. - 6 a.m.) vs. earlier time in the day, in the overall sample and according to different diabetes status, NDNS RP 2008-2017.

**Selected food groups**	**Overall**	**Healthy**	**Pre-diabetics**	**Undiagnosed diabetics**	**Diabetics**
Pudding	1.43 (1.06, 1.94)	1.55 (1.13, 2.15)	0.95 (0.17, 5.20)	1.82 (0.41, 8.03)	0.63 (0.15, 2.66)
Regular soft drink	1.72 (1.44, 2.05)	1.70 (1.41, 2.05)	1.78 (0.90, 3.48)	2.82 (1.24, 6.43)	1.36 (0.59, 3.10)
Sugar confectionery	1.84 (1.31, 2.59)	1.55 (1.08, 2.23)	2.13 (0.34, 13.24)	10.51 (2.35, 47.04)	5.94 (1.86, 19.00)
Chocolate	3.08 (2.62, 3.62)	2.98 (2.51, 3.54)	4.06 (1.98, 8.31)	2.41 (0.88, 6.60)	4.92 (2.38, 10.20)
Beer	7.26 (5.91, 8.92)	7.55 (6.04, 9.43)	4.42 (2.19, 8.95)	8.29 (3.70, 18.56)	5.82 (2.03, 16.68)
Ice cream	2.45 (1.84, 3.25)	2.52 (1.86, 3.41)	3.39 (0.77, 14.89)	1.07 (0.15, 7.77)	1.74 (0.57, 5.32)
Biscuit	1.90 (1.68, 2.16)	1.78 (1.55, 2.05)	3.25 (1.99, 5.28)	2.96 (1.43, 6.10)	2.33 (1.45, 3.77)
Crisp	1.49 (1.22, 1.82)	1.49 (1.21, 1.85)	2.21 (0.90. 5.41)	1.59 (0.43, 5.95)	0.89 (0.34, 2.33)

*Logistic regression models with GEE were adjusted for age, sex, body mass index, and social-economic levels*.

*NDNS RP: National Diet and Nutrition Survey Rolling Programme*.

The listed food groups were found to have higher odds to be consumed between 8 p.m. and 6 a.m. than earlier in the day. The main effect ORs (99% CIs) of consuming these foods in the evening/night were the following: for puddings 1.43 (1.06, 1.94), for regular soft drinks 1.72 (1.44, 2.05), for sugar confectioneries 1.84 (1.38, 2.69), for chocolates 3.08 (2.62, 3.62), for beers 7.26 (5.91, 8.92), for ice cream 2.45 (1.84, 3.25), for biscuits 1.90 (1.68, 2.16), for crisps 1.49 (1.22, 1.82) vs. earlier time. Opposite directions of the association for puddings were detected across diabetes status: the ORs (99% CIs) of consuming puddings at night time (8 p.m. or later) compared with earlier time were 1.55 (1.13, 2.15), 0.95 (0.17, 5.20), 1.82 (0.41, 8.03), and 0.63 (0.15, 2.66) for healthy, prediabetic, undiagnosed diabetic, and diabetic participants, respectively. Furthermore, undiagnosed diabetic patients were found to have particularly high odds of consuming regular soft drinks (OR: 2.82; 99% CI: 1.24, 6.43), and sugar confectioneries (OR: 10.61; 99%CI: 2.35, 47.04) during night-time in comparison with participants with other diabetes status. The same models were also used to estimate the ORs of consuming the selected food groups comparing participants with different diabetes status during either daytime (earlier than 8 p.m.) or nighttime (between 8 p.m. and 6 a.m.). Results are given in Supplementary Table 2.

## Discussion

The present study described the potential relationships between food groups and time of their consumption in a representative sample from the NDNS RP. Many unhealthy foods that emerged from CA were found to be more likely to be consumed after 8 p.m. These included alcoholic/sweetened beverages, chocolates, and other foods rich in added sugars and saturated fats, such as biscuits and ice cream. Foods chosen in the evening/night time slots tend to be highly processed and easily accessible. Specifically, undiagnosed patients might be at a higher risk of worsening their condition as they were found to have higher odds of consuming less healthy foods after 8 p.m. (sugar confectioneries, regular soft drinks) in comparison with diabetics and non-diabetics. Those foods might need to be targeted when designing an intervention for those who might be at risk of being diabetics.

These findings are concerning considering previous research has indicated that the quality of macronutrient intake in the evening is likely to influence fasting glucose levels and glycemic response to subsequent meals in the morning (32). One prospective study reported women who ate later than 9 p.m. had 1.51 times (95% CI 1.16 to 1.93) higher 5-year risk of developing prediabetes/diabetes than those having their last eating episode between 4 and 9 p.m. (33). More recently, a randomized controlled trial indicated that consuming carbohydrates at dinner irrespective of glycemic index raised postprandial glucose response to breakfast producing what is known as a second meal effect (34). Similar observations have been made by Nitta et al. who observed that eating sweets or snacks post-dinner worsened glycemic excursions in the evening and at subsequent breakfast (35). Added to this is evidence that suggests that the late-night dinners induce post prandial hyperglycemia in patients with type 2 diabetes and that interventions at these eating occasions can result in a profound impact on post-prandial glycemia. On the balance of this evidence, targeting and improving the timing and quality of foods in evening eating occasions provides a unique opportunity to design intervention for those who might be at risk of being diabetics.

A compelling finding of our study is the observation that diabetes patients were found to be potentially controlling their choice of food groups such as avoiding puddings at night. However, higher odds of consuming alcoholic beverages and energy condensed foods, such as chocolates and sugar confectioneries, at night among individuals with diabetes suggest that their food choice might need further modifications. Food intake late in the night is in misalignment with the circadian rhythm of the insulin response, which may cause greater glycemic exposure and elevated HbA1c levels even for healthy individuals (33). Disrupted timing of food intake, overeating in the evening, unhealthy food chosen at a later time in the day can result in poor glucose control and increase the likelihood of diabetic complications (4, 36–38). Assessing the relationships between food groups and timing of eating by diabetes status can be considered as a first step toward identifying specific public health targets for behavior change/intervention. This is important as most current public health strategies and dietary recommendations do not provide targeted advice that takes into consideration specific eating occasions while targeted advice is more likely to result in sustainable behavioral change. Our findings are consistent with previous evidence that has found that both sweetened and alcoholic beverages are responsible for a large portion of energy consumption at night in other populations (39).

However, an important limitation in this study is the cross-sectional study design. Our findings do not indicate whether it would be better for individual health to consume unhealthy foods later or earlier in the day, which should be clarified through purposedly designed intervention studies in the future. Some of the findings, such as higher consumption of alcoholic beverages at night are already known. However, the facts that certain snacks were more likely to be consumed at night and even more frequently among undiagnosed diabetics are an important piece of public health evidence as these data are representative of national behavior across the UK. Furthermore, the inability to assess the temporal relationship between timing of food intake and diabetes status means that a cause-effect relationship between the time of unhealthy food intake and diabetes status cannot be established. Hence, further prospective studies are warranted to investigate the causal relationship between diabetes and both quality and timing of eating. In addition, the current study assumes that mis-reporting occurred equally amongst all eating occasions. This limitation has been reported by previous literature as an important methodological limitation of chrononutrition (40); indeed, further investigation would be warranted to assess the effect of differential misreporting on epidemiological studies in chrononutrition to suggest possible corrections, e.g., for differential under-reporting at different times of the day (e.g., main meals vs. snack times). Finally, we did not include variables indicating abdominal obesity and sedentary lifestyle, such as physical activity or waist circumference in the second step of the current analysis mainly due to missingness of the variables. The associations comparing food consumed later vs. earlier in the day presented in this study may be partly explained or mediated through a low level of physical activity and/or abdominal obesity especially among those who were unaware that they have diabetes: further investigations to assess the role of these variables in chrononutrition are also warranted.

## Conclusion

In summary, our study indicates that foods consumed in the evening/night time tend to be highly processed, easily accessible, and rich in added sugar or saturated fat, whatever the diabetic status is. Individuals with undiagnosed diabetes are more likely to consume specific unhealthy foods at night. The survey cross-sectional nature warrants further investigations by longitudinal cohort studies to establish the causal relationship between the time of eating unhealthy foods and diabetes.

## Data Availability Statement

Publicly available datasets were analyzed in this study. Original data used in this study can be accessed upon request to the UK Data Service (https://www.ukdataservice.ac.uk) for academic usage (Study Number: 6533).

## Ethics Statement

The studies involving human participants were reviewed and approved by the NDNS RP, funded by Public Health England and the UK Food Standards Agency, is registered with the ISRTCN registry under study ID ISRCTN17261407 and received ethical approval from the Oxfordshire Research Ethics Committee. The patients/participants provided their written informed consent to participate in this study.

## Author Contributions

CW, SA, and LP designed research and had primary responsibility for final content. CW and LP performed statistical analysis. All authors wrote the manuscript, read, and approved the final manuscript.

## Funding

This work was supported by Grant-in-Aid for Early-Career Scientists (Grant No. 19K20199 to CW) from the Japan Society for the Promotion of Science (JSPS).

## Conflict of Interest

The authors declare that the research was conducted in the absence of any commercial or financial relationships that could be construed as a potential conflict of interest.

## Publisher's Note

All claims expressed in this article are solely those of the authors and do not necessarily represent those of their affiliated organizations, or those of the publisher, the editors and the reviewers. Any product that may be evaluated in this article, or claim that may be made by its manufacturer, is not guaranteed or endorsed by the publisher.
