# Supplementary material for: Relationships Between Food Groups and Eating Time Slots According to Diabetes Status in Adults From the UK National Diet and Nutrition Survey (2008–2017)
Source: Front Nutr. 2021 Sep 30;8:692450. doi: 10.3389/fnut.2021.692450 (PMC8514704; doi:10.3389/fnut.2021.692450)
Supplement: Supplementary file 1 [file Data_Sheet_1.PDF]

Supplementary Material

SUPPLEMENTARY TABLE

Table S1. Contingency matrix of 60 standard foods by 7 time slots containing the percentages (numbers of data entries) in the NDNS RP (2008-2017).

Food Groups / Time Slots	6am to 9am	9am to 12am	12 noon to 2pm	2pm to 5pm	5pm to 8pm	8pm to 10pm	10pm to 6am	Total
Artificial sweeteners	24.13% (1814)	23.22% (1746)	12.09% (909)	14.91% (1121)	10.99% (826)	9.31% (700)	5.35% (402)	(7518)
Bacon and ham	4.98% (422)	16.95% (1435)	39.62% (3355)	10.66% (903)	21.45% (1816)	4.94% (418)	1.39% (118)	(8467)
Beef	0.10% (5)	0.96% (48)	17.97% (896)	10.15% (506)	58.17% (2901)	11.23% (560)	1.42% (71)	(4987)
Beer lager	0.37% (30)	0.72% (59)	6.56% (538)	10.96% (899)	25.19% (2065)	36.02% (2953)	20.19% (1655)	(8199)
Biscuits	6.23% (822)	17.79% (2348)	17.18% (2268)	21.31% (2813)	12.57% (1659)	15.86% (2093)	9.07% (1197)	(13200)
Brown bread	17.43% (1078)	18.63% (1152)	35.06% (2168)	8.94% (553)	11.68% (722)	5.52% (341)	2.73% (169)	(6183)
Burgers/kebabs	0.00% (0)	2.56% (24)	22.15% (208)	16.72% (157)	44.62% (419)	10.44% (98)	3.51% (33)	(939)
Butter	15.75% (1607)	17.45% (1780)	24.11% (2460)	10.74% (1096)	21.44% (2188)	7.69% (785)	2.81% (287)	(10203)
Cakes & pastries	4.69% (366)	15.18% (1185)	18.52% (1446)	23.28% (1817)	21.88% (1708)	11.80% (921)	4.65% (363)	(7806)
Cheese	2.67% (293)	7.15% (785)	34.71% (3812)	11.80% (1296)	28.17% (3094)	12.12% (1331)	3.39% (372)	(10983)
Chicken/turkey	0.35% (31)	2.23% (198)	23.95% (2123)	12.60% (1117)	46.27% (4101)	12.08% (1071)	2.50% (222)	(8863)
Chips	0.56% (38)	3.20% (216)	18.86% (1273)	12.96% (875)	51.44% (3472)	10.70% (722)	2.27% (153)	(6749)
Chocolate	1.74% (113)	10.33% (671)	14.67% (953)	22.36% (1452)	16.09% (1045)	25.33% (1645)	9.48% (616)	(6495)
Coated chicken	0.09% (1)	1.54% (18)	17.18% (201)	15.13% (177)	50.51% (591)	12.14% (142)	3.42% (40)	(1170)
Commercial toddlers foods	7.58% (5)	4.55% (3)	33.33% (22)	13.64% (9)	30.30% (20)	7.58% (5)	3.03% (2)	(66)
Crisps	0.49% (28)	9.27% (525)	31.59% (1789)	18.47% (1046)	15.18% (860)	16.67% (944)	8.33% (472)	(5664)
Diet soft drinks	5.38% (761)	9.53% (1349)	16.40% (2322)	15.70% (2222)	26.97% (3818)	16.33% (2311)	9.69% (1372)	(14155)
Dietary supplements	71.61% (9321)	18.87% (2456)	2.09% (272)	1.13% (147)	1.79% (233)	1.84% (239)	2.67% (348)	(13016)
Eggs	10.86% (820)	20.86% (1576)	24.36% (1840)	9.89% (747)	25.47% (1924)	7.04% (532)	1.52% (115)	(7554)
Fruit	15.63% (5300)	17.58% (5959)	21.42% (7262)	14.46% (4903)	17.45% (5916)	10.33% (3501)	3.13% (1062)	(33903)
Fruit juice	27.53% (1916)	17.40% (1211)	14.71% (1024)	9.87% (687)	18.49% (1287)	8.22% (572)	3.78% (263)	(6960)
High fiber cereals	62.33% (5120)	29.88% (2455)	2.17% (178)	1.03% (85)	0.97% (80)	1.56% (128)	2.06% (169)	(8215)
Ice cream	0.06% (1)	0.88% (16)	11.34% (206)	16.08% (292)	42.35% (769)	24.01% (436)	5.29% (96)	(1816)
Jams & spreads	24.32% (9231)	23.62% (8965)	12.21% (4636)	13.02% (4942)	13.13% (4983)	8.71% (3306)	5.00% (1897)	(37960)
Lamb	0.08% (1)	0.32% (4)	18.94% (237)	12.71% (159)	49.16% (615)	16.31% (204)	2.48% (31)	(1251)
Liver	4.41% (20)	8.15% (37)	34.36% (156)	11.67% (53)	29.07% (132)	11.01% (50)	1.32% (6)	(454)
Low fiber cereals	55.22% (2376)	32.16% (1384)	3.25% (140)	1.56% (67)	1.81% (78)	2.32% (100)	3.67% (158)	(4303)
Low fat spreads	18.31% (669)	19.13% (699)	29.06% (1062)	8.05% (294)	17.21% (629)	5.61% (205)	2.63% (96)	(3654)
Low-fat milk	30.48% (385)	21.62% (273)	9.82% (124)	12.83% (162)	11.64% (147)	8.16% (103)	5.46% (69)	(1263)
Margarine	3.19% (279)	6.91% (604)	18.53% (1620)	10.41% (910)	44.29% (3872)	14.54% (1271)	2.13% (186)	(8742)
Meat pastries	1.01% (20)	6.72% (133)	32.49% (643)	15.31% (303)	32.84% (650)	8.79% (174)	2.83% (56)	(1979)
Misc./Vending	3.65% (1775)	6.51% (3164)	23.87% (11598)	11.35% (5517)	38.57% (18742)	12.52% (6086)	3.53% (1715)	(48597)
Nuts and seeds	20.88% (1307)	17.26% (1080)	14.87% (931)	13.26% (830)	16.71% (1046)	11.81% (739)	5.21% (326)	(6259)
Oily fish	1.61% (42)	3.98% (104)	29.46% (769)	11.15% (291)	41.53% (1084)	10.84% (283)	1.42% (37)	(2610)
Other bread	17.06% (159)	16.09% (150)	34.66% (323)	10.52% (98)	13.73% (128)	6.01% (56)	1.93% (18)	(932)
Other meat	4.77% (75)	10.18% (160)	33.35% (524)	11.33% (178)	28.39% (446)	9.48% (149)	2.48% (39)	(1571)
Other milk cream	18.29% (1208)	16.46% (1087)	13.81% (912)	12.73% (841)	23.85% (1575)	11.02% (728)	3.85% (254)	(6605)
Other white fish	0.51% (19)	3.46% (128)	33.00% (1222)	10.26% (380)	37.51% (1389)	13.04% (483)	2.21% (82)	(3703)
Pasta & rice and other cereals	7.61% (1396)	5.90% (1083)	17.03% (3126)	10.10% (1853)	42.97% (7887)	13.51% (2479)	2.88% (529)	(18353)
Polyunsaturated margarine	3.59% (114)	6.73% (214)	17.40% (553)	9.88% (314)	46.30% (1472)	13.72% (436)	2.39% (76)	(3179)
Pork	0.44% (8)	1.69% (31)	18.12% (332)	12.01% (220)	54.09% (991)	12.55% (230)	1.09% (20)	(1832)
Other potatos	0.21% (21)	1.02% (103)	19.24% (1946)	10.60% (1072)	59.04% (5971)	9.04% (914)	0.85% (86)	(10113)
Puddings	0.87% (20)	3.10% (71)	22.00% (504)	12.79% (293)	42.69% (978)	14.62% (335)	3.93% (90)	(2291)
Regular soft drinks	5.23% (596)	9.39% (1069)	18.57% (2114)	16.39% (1866)	24.84% (2829)	16.19% (1844)	9.39% (1069)	(11387)
Salad and raw vegetables	0.86% (285)	3.78% (1257)	35.17% (11697)	11.48% (3820)	36.37% (12098)	10.65% (3544)	1.69% (562)	(33263)
Sausages	4.96% (150)	19.27% (583)	18.71% (566)	10.18% (308)	38.05% (1151)	7.64% (231)	1.19% (36)	(3025)
Semi-skimmed milk	26.43% (15225)	23.64% (13622)	11.34% (6531)	13.81% (7957)	11.02% (6349)	8.49% (4891)	5.27% (3036)	(57611)
Skimmed (fat-free) Milk	28.72% (2781)	21.41% (2073)	10.62% (1028)	13.50% (1307)	10.84% (1050)	8.97% (869)	5.94% (575)	(9683)
Smoothies	21.90% (30)	21.90% (30)	24.09% (33)	13.87% (19)	5.84% (8)	9.49% (13)	2.92% (4)	(137)
Spirits and liqueurs	0.22% (6)	0.96% (26)	2.67% (72)	5.49% (148)	22.28% (601)	38.36% (1035)	30.02% (810)	(2698)
Spreads less-fat	15.37% (1940)	20.52% (2590)	28.87% (3643)	8.58% (1083)	17.12% (2161)	6.51% (822)	3.02% (381)	(12620)
Sugar confectionery	4.62% (84)	12.53% (228)	12.80% (233)	26.43% (481)	15.66% (285)	19.29% (351)	8.68% (158)	(1820)
Tea/coffee/water	18.70% (28448)	21.28% (32367)	13.76% (20926)	15.55% (23657)	13.73% (20881)	9.70% (14758)	7.28% (11071)	(152108)
Vegetables not raw	0.78% (399)	2.52% (1293)	20.65% (10599)	9.83% (5042)	53.37% (27389)	11.62% (5963)	1.23% (632)	(51317)
White bread	12.92% (2382)	19.14% (3529)	30.06% (5542)	9.81% (1808)	18.02% (3322)	7.14% (1317)	2.90% (534)	(18434)
White fish, shellfish	0.13% (2)	0.88% (14)	19.35% (309)	9.33% (149)	59.42% (949)	10.14% (162)	0.75% (12)	(1597)
Whole milk	24.26% (3306)	22.56% (3075)	11.56% (1575)	12.27% (1672)	13.39% (1825)	9.53% (1299)	6.43% (876)	(13628)
Wine	0.04% (3)	0.42% (29)	5.20% (362)	5.40% (376)	38.61% (2690)	39.53% (2754)	10.81% (753)	(6967)
Whole meal bread	19.51% (1403)	18.05% (1298)	33.62% (2418)	8.09% (582)	13.54% (974)	4.78% (344)	2.42% (174)	(7193)
Yogurt	16.04% (1087)	12.43% (842)	24.38% (1652)	9.36% (634)	23.72% (1607)	10.92% (740)	3.16% (214)	(6776)
Total	14.30% (107144)	14.77% (110614)	18.45% (138183)	12.63% (94606)	24.10% (180498)	10.91% (81716)	4.84% (36265)	(749026)

NDNS RP: National Diet and Nutrition Survey Rolling Programme.

Table S2. Odds ratio (OR, 99% confidence intervals) for food groups eaten by different diabetes status taking healthy pariticipants as the reference group at Daytime or Nighttime, NDNS RP 2008-2017.

		Earlier than 8 pm		8 pm to 6 am	
		OR	99% CI	OR	99% CI
Pudding	Pre-diabetics	1.19	0.63, 2.24	0.73	1.06, 1.94
	Undiagnosed diabetics	0.90	0.46, 1.81	1.06	0.28, 3.99
	Diabetics	1.46	0.90, 2.41	0.59	0.15, 2.36
	Healthy	1	–	1	–
Regular soft drink	Pre-diabetics	0.87	0.42, 1.82	0.91	0.45, 1.83
	Undiagnosed diabetics	1.12	0.49, 2.53	1.85	0.62, 5.57
	Diabetics	0.86	0.48, 1.54	0.69	0.29, 1.57
	Healthy	1	–	1	–
Sugar confectionery	Pre-diabetics	0.56	0.18, 1.71	0.77	0.17, 3.42
	Undiagnosed diabetics	0.44	0.12, 1.56	2.98	0.52, 16.97
	Diabetics	0.63	0.25, 1.60	2.42	0.76, 7.69
	Healthy	1	–	1	–
Chocolate	Pre-diabetics	0.94	0.53, 1.65	1.28	0.69, 2.37
	Undiagnosed diabetics	0.93	0.46, 1.89	0.75	0.34, 1.68
	Diabetics	0.58	0.34, 0.99	0.96	0.54, 1.69
	Healthy	1	–	1	–
Beer	Pre-diabetics	1.21	0.69, 2.11	0.71	0.36, 1.39
	Undiagnosed diabetics	1.09	0.49, 2.39	1.19	0.51, 2.84
	Diabetics	0.86	0.29, 2.56	0.66	0.28, 1.57
	Healthy	1	–	1	–
Ice cream	Pre-diabetics	0.85	0.32, 2.26	1.14	0.23, 5.72
	Undiagnosed diabetics	1.14	0.49, 2.58	0.48	0.08, 2.92
	Diabetics	0.96	0.54, 1.73	0.67	0.19, 2.28
	Healthy	1	–	1	–
Biscuit	Pre-diabetics	0.81	0.58, 1.14	1.48	0.94, 2.32
	Undiagnosed diabetics	0.78	049, 1.22	1.29	0.65, 2.54
	Diabetics	0.94	0.69, 1.28	1.23	0.77, 1.97
	Healthy	1	–	1	–
Crisp	Pre-diabetics	1.01	0.61, 1.65	1.48	0.70, 3.14
	Undiagnosed diabetics	0.91	0.45, 1.84	0.97	0.25, 3.77
	Diabetics	1.07	0.72, 1.59	0.64	0.25, 1.62
	Healthy	1	–	1	–

Logistic regression models with GEE were adjusted for age, sex, body mass index, and social-economic levels.
NDNS RP: National Diet and Nutrition Survey Rolling Programme
